# Molecular epidemiology of respiratory syncytial virus among children and adults in India 2016 to 2018

**DOI:** 10.1007/s11262-021-01859-4

**Published:** 2021-09-15

**Authors:** Suresh S. Bandla, Santhosha Devadiga, Rushil Bhatt, Oliver C. Dsa, Arunkumar Govindakarnavar

**Affiliations:** 1 Manipal Institute of Virology (MIV), Manipal Academy of Higher Education (MAHE), Manipal, Karnataka 576104 India; 2 Present Address: Vaikathu, Athrady, Udupi, Karnataka 576107 India

**Keywords:** Respiratory syncytial virus, G gene, Human orthopneumovirus, Amino acid substitution, Molecular epidemiology, India

## Abstract

Respiratory syncytial virus (RSV) is a common cause of respiratory tract infections among children less than 5 years of age and the elderly. This study intended to determine the circulating genotypes of RSV among severe acute respiratory illness (SARI) cases during the period 2016–2018 in India, among hospitalized acute febrile illness cases of age ranging from 1 to 65 years. Throat/nasopharyngeal swab samples were subjected for testing RSV and subgroups by real-time reverse transcriptase polymerase chain reaction (RT-PCR), further sequencing and phylogenetic analysis were performed for the second hypervariable region of the G gene. RSV-A and B subtypes co-circulated during the years 2016, 2017, and 2018, with RSV-A as the dominant subtype in 2016, and RSV-B as the dominant subgroup in 2017 and 2018. Phylogenetic analysis revealed that the circulating genotypes of RSV were GA2 (16/16), of RSV-A, and GB5 (23/23) of RSV-B in the South, North, and Northeast region of India during the period between 2016 and 2018. Here we report the first study comprising the distribution of RSV-A and B genotypes in the different geographic regions of India among children and adults during the year 2016 to 2018. We also report GA2.3.7 lineage of GA2 genotype for the first time in India to the best of our knowledge.

**Supplementary Information:**

The online version contains supplementary material available at 10.1007/s11262-021-01859-4.

## Introduction

Acute lower respiratory tract infections are the major cause of hospitalization and death among children less than 5 years of age, with RSV being an important viral pathogen causing the infection worldwide [1, 2]. Previous studies estimate that annually about 33.1 million RSV infections occur, among which about 3.2 million are hospitalized cases with around 59,600 deaths occurring in children younger than 5 years [3]. Almost every child by the age of 2 years gets infected with RSV infection, and primary infection is rarely found to be asymptomatic [4]. Reinfections are common among individuals of any age group, RSV infections even lead to morbidity and mortality among the elderly [5]. The incubation period of RSV in human infection ranges from 3 to 5 days [6]. The common symptoms of RSV are nasal congestion, coryza, nonproductive cough, and mild grade fever that may lead to the involvement of the lower respiratory tract causing bronchiolitis, pneumonia, and tracheobronchitis [7–9]*.* The attributable mortality rate estimated due to RSV infections is around 0.86 per 1000 live births [10]. RSV infections were usually observed from March to October months in Southern Hemisphere, whereas during September to May months in Northern Hemisphere [11]*.* In India, RSV infections are observed during monsoons, which peaks during September and October months [12–15].

RSV genome size is approximately 15,222 nucleotides that comprise 10 genes encoding 11 proteins [16]*.* Based on the -antibody cross-reactivity patterns of the G glycoprotein [17], RSV has been classified into A and B subgroups that were further classified into 20 RSV-A genotypes: (GA1-7, SAA1-2, NA1-4, ON1-2, LBA1-2, CB-A, and TN1-2) and 39 RSV-B genotypes: (GB1–13, THB, BA1–14, BA-LY, BA-C, CB1, BA-CCA, BA-CCB, SAB1–4, and URU1–2) respectively [17–20]. Recently the RSV genotypes were reclassified for both the RSV-A and RSV-B subgroups. The reclassification of the RSV-A subgroup reported 3 genotypes (GA1-3) along with subgenotypes and lineages, whereas the reclassification of the RSV-B subgroup reported 7 genotypes (GB1-7) along with subgenotypes and lineages [21]. The current circulating genotypes among group A are GA2 and GA3 genotypes, and GB5 and GB7 genotypes among group B, worldwide [21]*.* In India, GA2.3.0, GA2, GA3.0.3, GA2.3.3, GA2.3.1, and GA2.3.5 genotypes for subgroup A, and GB2, GB5.0.2, GB5.0.5c, and GB5.0.2 genotypes for subgroup B have been reported [14, 22, 23]*.*

RSV can become a vaccine-preventable disease if an effective vaccine is developed against it; however, there are several vaccination strategies in advanced clinical phases for protection against severe RSV disease. For developing an effective vaccine against RSV there is a need to understand the circulating genotypes. However, there is limited data available on the molecular epidemiology of RSV among children and adults in the different geographical regions of India. Here we report a study to understand the molecular epidemiology of RSV in different geographical regions of India during the April 2016–September 2018 time period.

## Material and methods

### Study population

**Fig. 1:**
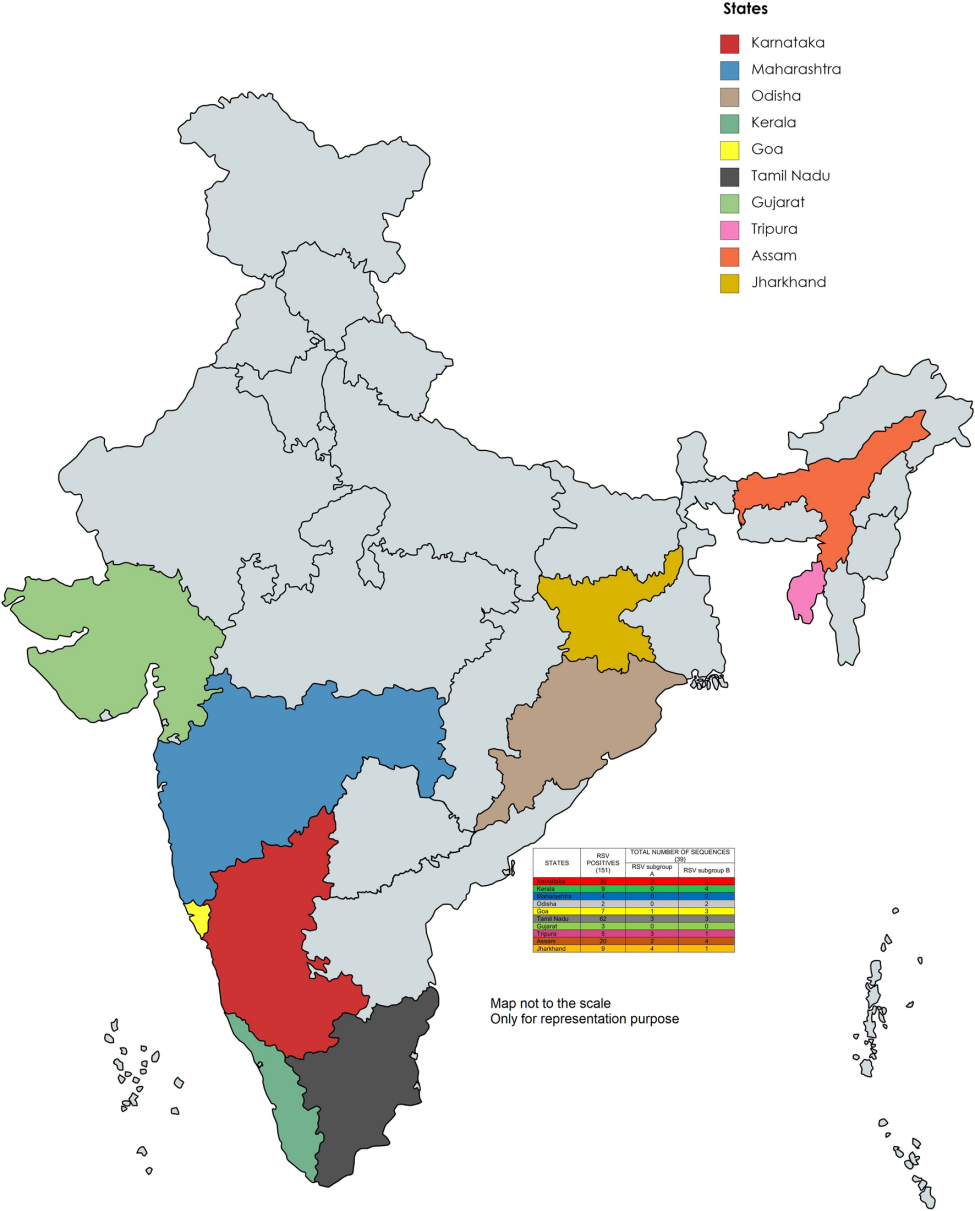
Map showing ten states from which samples were represented under the AFI surveillance conducted by MIV in India. The number of sequences obtained from each state was shown in the table next to the map

A total of 151 RSV-positive archived samples have been included in this study and cases from 10 states of India; Karnataka, Kerala, Assam, Goa, Gujarat, Maharashtra, Jharkhand, Tripura, Tamil Nadu and Odisha depicted in (Fig. 1) under Acute Febrile Illness (AFI) surveillance study conducted by Manipal Institute of Virology (MIV), with a case definition of patients admitted to hospital with fever (≥ 38 ℃) of age between 1 and 65 years were recruited and samples were tested for various viral pathogens including RSV, bacterial and parasite agents by molecular and serological methods [24]. All the inpatients admitted in the hospitals fulfilling the inclusion criteria of the case definition were included in the surveillance study The surveillance study was conducted for a duration of 5 years from 2014 to 2019 [24]. The total number of the AFI samples that were obtained annually during the study period is as follows: 2016–12,414, 2017–20,359, 2018–4954. RSV-positive archived respiratory specimens of the AFI study were used in this study. The current study was reviewed and approved by the Institutional Ethical Committee, Manipal Academy of Higher Education (IEC No: UEC/32/2013-14, MUEC/Renewal-08/2017). Cases were recruited after obtaining Informed consent as part of the AFI study.

### Real-time RT-PCR for subgrouping

RNA was extracted from 150 μl of 151 RT-PCR RSV-positive throat swab samples using QIAamp® Viral RNA mini kit (QIAGEN, Hilden, Germany) as per manufacturer’s instructions. Real-time RT-PCR was performed using primers targeting the N gene that was published by Hu et al. using AgPath-ID™ One-step RT-PCR kit (Applied Biosystems, Foster City, USA) to determine the RSV subgroups of the samples [25]*.* The reaction was performed in a QuantStudio™ 5 PCR system (Applied Biosystems, Foster City, USA). The cycling conditions for the PCR reaction were as follows: reverse transcription at 50 °C for 30 min, initial denaturation at 95 °C for 15 min, followed by 40 cycles of denaturation and extension at 95 °C for 15 s, 58 °C for 30 s respectively.

### Genotyping of RSV and sequencing

Sixty-nine samples were then selected by a purposive sampling method based on the state (place), the year during the illness, and age of the patients for the determination of the genotypes. As the major number of cases were from two states, to remove the bias, 10 samples have been selected from each state based on the year during the illness. All the samples were included for genotyping from the states with samples that are less than 10 in number. Seminested PCR was carried out by using the primers targeting the second hypervariable region of the G gene that were published Parveen et al. using the AgPath-ID™ One-step RT-PCR kit (Applied Biosystems, Foster City, USA) [22]*.* The reactions were performed in a ProFlex™ 5 PCR system (Applied Biosystems, Foster City, USA). The amplified products were purified using the GenElute™ Gel purification Kit (Sigma Aldrich, Missouri, USA) according to the manufacturer’s instructions. The purified products were sequenced using the Big Dye Terminator kit v3.1 cycle sequencing kit (Applied Biosystems, Foster City, USA) using ABI 3500xl genetic analyzer (Applied Biosystems, Foster City, USA).

### Sequence analysis and phylogeny

The sequences obtained consisted of partial regions of the second hypervariable region of the G gene including *N*-terminal of F gene. The genome region ranging from 5419 to 5643 nucleotides in reference to the sequence Acc. No.: JX627336 of RSV-A subgroup, and the genome region ranging from 5349 to 5618 nucleotides in reference to the sequence Acc. No. KF640637 of the RSV-B subgroup was used for the construction of the phylogenetic tree and amino acid alignment. The published sequences of the second hypervariable region of the G gene of RSV from different geographical regions of the world were downloaded from the GenBank of NCBI (National Centre for Biotechnology Information) database. Multiple sequence alignments of study sequences for the second hypervariable region of the G gene with known RSV genotypes that were recently classified by Goya et al. were carried out using MUSCLE (Multiple Sequence Comparison by Log Expectation) algorithms in MEGA X version 10.0.5 [21]. The phylogenetic trees of nucleotide sequences were constructed separately for RSV-A and RSV-B subgroups with Bayesian Information criterion using the Maximum Likelihood method. The suitable substitution model for RSV-A sequences was GTR + G substitution model and the same was used for the construction of the phylogenetic tree, whereas HKY85 + G was the suitable substitution model for RSV-B sequences and the same was used for construction of the phylogenetic tree with a support value of 1000 bootstrap replicates in PhyML software (version 3.0). The trees were visualized and midpoint trees were constructed using Figtree software. A total of 16 RSV-A and 23 RSV-B sequences of the second hypervariable region of the G gene were deposited in the GenBank with the accession number MN463622 to MN463637 and MN463638 to MN463660 respectively.

### P-distance analysis

The intergenotypic and intragenotypic p-distances of the second hypervariable region of G gene sequences of RSV-A and RSV-B subgroups were calculated separately for each of the subgroups. The p-distance matrices were generated using MEGA X version 10.0.5 software.

### Data analysis

Sociodemographic factors, clinical features, and laboratory parameters of the RSV cases were obtained from the AFI study database and analyzed using SPSS 15.0 for Windows software (SPSS™ Inc, Chicago, IL, USA). For analysis of continuous variables, one-way anova test and analysis of categorical variables, the chi-square test was used. A *p* value of < 0.05 was set as the level of statistical significance.

## Results

### Description of the study population

Among AFI cases recruited, a total of 151 samples tested positive for RSV by RT-PCR during the period 2016 to 2018, out of 10 states, the major number of cases, 62 cases (41.1%) were from Tamil Nadu, 30 (19.9%) from Karnataka, and 20 (13.2%) from Assam were represented in (Table 1). The mean age of the study population is 22.2 years. The positivity rate of the RSV annually for each year during the AFI study period is 0.6% (73/12414) in 2016, 0.2% (42/20359) in 2017, and 0.7% (36/4954) in 2018. Out of the study population, 79 (52.3%) patients were males and 72 (47.7%) were females. Most of the RSV infected cases were of low and middle socioeconomic status 75 (49.7%) and 74 (49%) respectively. The mean duration of hospital stay was observed to be 3.64 days. The frequencies of symptoms and signs among RSV cases were summarized in (Table 1). RSV cases were presented with symptoms like cough (94%), general weakness (89.4%), and coryza (78.1%). Coryza (*p* = 0.015), headache (*p* < 0.001), night sweats (*p* = 0.035) and joint pain (*p* = 0.036) were significantly associated with the RSV infection among adults and children (Supplementary Table 1). Mean ESR and CRP levels were observed to be elevated.

**Table 1:** Demographic, epidemiological, and clinical characteristics of RSV-A and RSV-B subgroups included in the study

Variables	*N*	Present	%	RSV(*n* = 123)		*p* value
				A	B	
Gender						
Male	151	79	52.3	27	37	–
Female	151	72	47.7	18	41	–
Socio Economic Status (Modified Udai Pareek scale)						
Low	151	75	49.7	20	42	–
Middle	151	74	49	24	35	–
High	151	2	1.3	1	1	–
State						
Assam	151	20	13.2	9	5	–
Goa	151	7	4.6	2	3	–
Gujarat	151	3	2	1	2	–
Karnataka	151	30	19.9	11	12	–
Kerala	151	9	6	1	8	–
Maharashtra	151	4	2.6	0	4	–
Odisha	151	2	1.3	0	2	–
Tamil Nadu	151	62	41.1	12	38	–
Tripura	151	5	3.3	3	2	–
Jharkhand	151	9	6	6	2	–
Year						
2016	151	73	48.3	38	13	–
2017	151	42	27.8	2	36	–
2018	151	36	23.8	5	29	–
Clinical symptoms						
Cough	151	142	94	38	77	**0.005**
General weakness	151	135	89.4	39	73	0.522
Coryza	151	118	78.1	33	62	0.736
Headache	151	111	73.5	34	57	0.466
Myalgia	151	102	67.5	30	52	0.842
Chills	151	93	61.6	28	46	0.461
Night sweats	151	62	41.1	23	27	0.073
Sore throat	151	60	29.7	15	34	0.239
Joint pain	151	61	40.4	16	32	0.477
Breathlessness	151	23	15.2	7	13	0.848
Chest pain	151	20	13.2	6	12	0.735

Bold value indicates statistical significance (*p* value < 0.05)

### RSV subgrouping

**Fig. 2:**
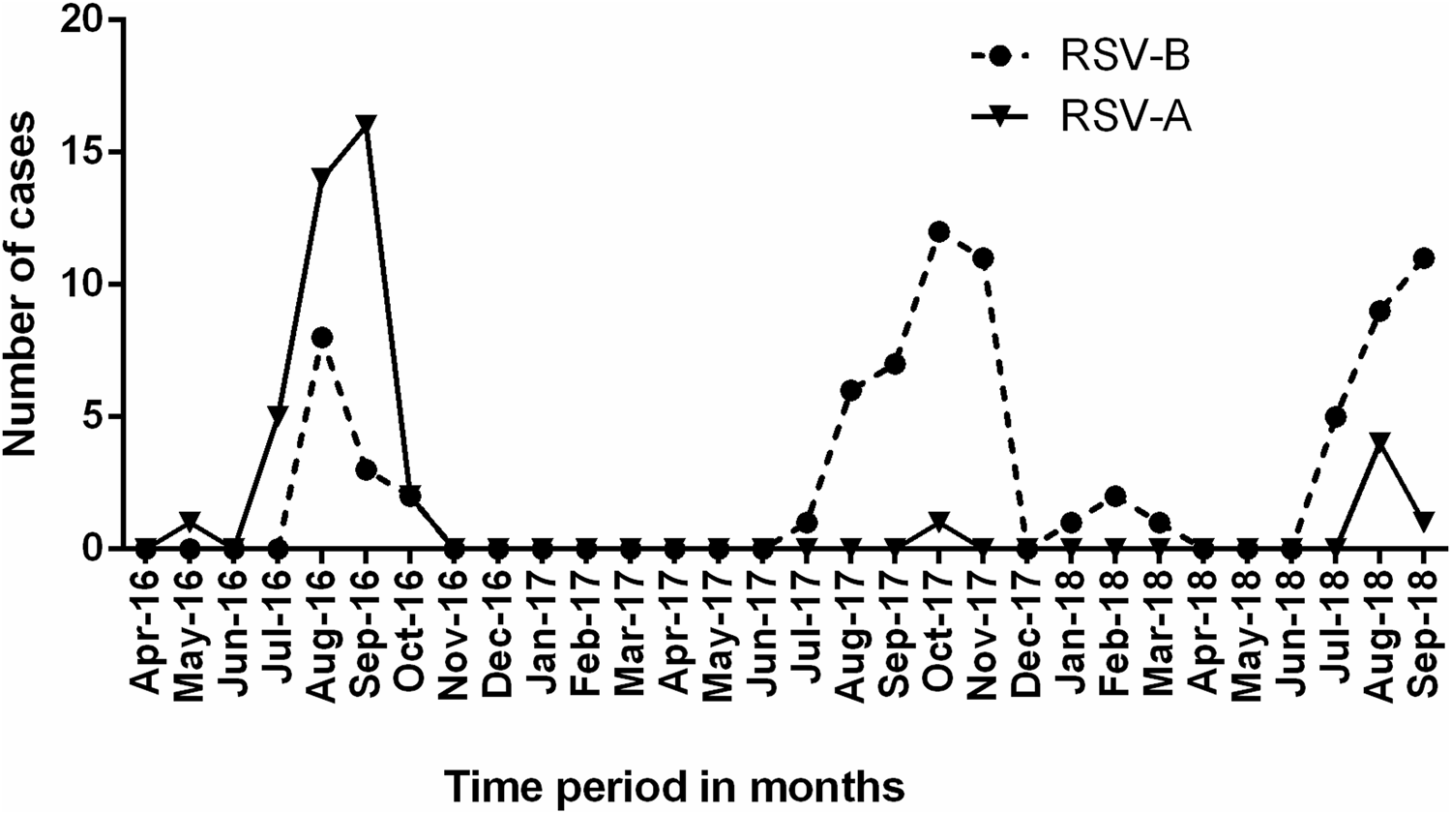
Seasonal distribution of the respiratory syncytial virus (RSV) subgroups in India during the period 2016–2018. Number of RSV cases were mentioned on *Y*-axis and time period mentioned on *X*-axis, the line graph represents the Respiratory Syncytial Virus-A (RSV-A) subgroup and the dotted line graph represents the Respiratory Syncytial Virus-B (RSV-B) subgroup

A total of 151 samples were subjected to RSV subgrouping of which 45 (29.8%) were of RSV-A subgroup and 78 (51.7%) were of RSV-B subgroup, remaining 28 (18.5%) samples could not be subgrouped using real-time PCR. 45 RSV-A subgroup samples from each state were as follows: Tamil Nadu (*n* = 12), Karnataka (*n* = 11), Assam (*n* = 9), Jharkhand (*n* = 6), Tripura (*n* = 3), Goa (*n* = 2), and Gujarat (*n* = 1). 78 RSV-B subgroup samples from each state were as follows: Tamil Nadu (*n* = 38), Karnataka (*n* = 12), Kerala (*n* = 8), Assam (*n* = 5), Maharashtra (*n* = 4), Goa (*n* = 3), Gujarat (*n* = 2), Odisha (*n* = 2), Tripura (*n* = 2), and Jharkhand (*n* = 2). Cough (*p* = 0.005) was significantly associated with the RSV-A and RSV-B subgroup infections (Table 1). The mean hematological parameters total leucocyte count, differential neutrophil count, and differential lymphocyte count were found within the normal limit range among the patients with RSV-A and B subgroup infection, and even among the RSV infected adults and children (Table 2 & Supplementary Table 2). The seasonality of RSV shows a clear circulation pattern of RSV each year between June and October. This circulation pattern can be correlated with the monsoon season in these regions. As can be seen in (Fig. 2) there is a co-circulation of RSV-A and B subgroups in all the three years 2016, 2017, and 2018, with RSV-A as the dominant subgroup in 2016, and RSV-B as the dominant subgroup in 2017 and 2018.

**Table 2:** Laboratory parameters of RSV cases included in the study

Laboratory parameters at admission	All	RSV		*p* value
		A	B	
Mean hospital stay (in days) (*n* = 151)	3.64	3.60	3.67	0.853
Mean Age years (*n* = 151)	22.2	19.4	23.4	0.498
Mean Hemoglobin levels gm/dl (*n* = 112)	12.1	12.2	11.8	0.166
Mean Platelet count × 10^3^/µl (*n* = 110)	224	232	218	0.693
Mean Total Leucocyte count /mm^3^ (*n* = 111)	6849	7038	7063	0.202
Mean Differential Neutrophil percentage (*n* = 105)	61.3	61.7	61	0.972
Mean Differential Lymphocyte percentage (*n* = 105)	31.6	30.9	32	0.926
Mean Differential Eosinophil percentage (*n* = 105)	4.2	4.7	4	0.483
Mean Differential Monocyte percentage (*n* = 105)	3.2	3	3.4	0.401
Mean Differential Basophil Percentage (*n* = 105)	1.1	0	1	0.143
Mean Erythrocyte Sedimentation Rate mm/h (*n* = 22)	31.8	29.2	30.6	0.836
Mean Urea levels mg/dl (*n* = 49)	21.5	20.5	21.4	0.521
Mean Creatinine levels mg/dl (*n* = 48)	0.8	0.8	0.7	0.107
Mean Total Bilirubin levels mg/dl (*n* = 71)	0.5	0.6	0.5	0.713
Mean Direct Bilirubin levels mg/dl (*n* = 41)	0.2	0.2	0.2	0.845
Mean Alkaline Phosphatase levels IU/L (*n* = 46)	392.4	442.9	346.2	0.478
Mean C-Reactive protein levels mg/l (*n* = 69)	15.6	14.5	15.8	0.940
Mean Aspartate aminotransferase levels IU/L (*n* = 71)	46.3	45.7	47.6	0.925
Mean Alanine aminotransferase levels IU/L (*n* = 71)	31.3	36.5	25	0.172

### Nucleotide sequencing

A total of 39 sequences second hypervariable region of the G gene were obtained. Sequencing results reconfirmed the results of subgrouping done by real-time PCR. 16 RSV-A sequences from each state were as follows: Jharkhand (*n* = 4), Tripura (*n* = 3), Tamil Nadu (*n* = 3), Karnataka (*n* = 3), Assam (*n* = 2), and Goa (*n* = 1) were analyzed and used for the construction of the phylogenetic tree. 23 RSV-B sequences from each state were as follows: Jharkhand (*n* = 1), Tripura (*n* = 1), Tamil Nadu (*n* = 3), Karnataka (*n* = 3), Assam (*n* = 4), Goa (*n* = 3), Odisha (*n* = 2), Kerala (*n* = 4), and Maharashtra (*n* = 2) were analyzed and used for the construction of the phylogenetic tree.

### Phylogenetic, amino acid, and p-distance analyses

**Fig. 3:**
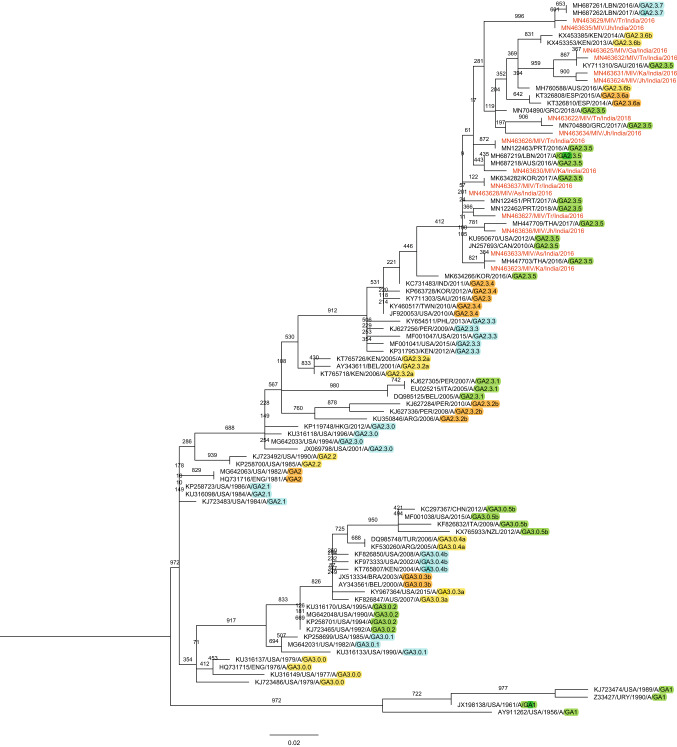
Mid-rooted phylogenetic tree of RSV-A subgroup. Mid-rooted phylogenetic tree of the second hypervariable region of the RSV-A G protein gene constructed by using Maximum Likelihood method, by GTR + G substitution model with 1000 bootstrap replicates in PhyML software. Study sequences are displayed in red. The reference sequences used to construct the tree were downloaded from the GenBank database. The two lettered words in the labels of the MIV isolates represent the state names, where: T* n* = Tamil Nadu, Ka = Karnataka, Jh = Jharkhand, Ga = Goa, Tr = Tripura, and As = Assam. The grid lines over the tree represent the patristic distance where the distance between each grid is 0.005. The names of the genotypes and lineages were highlighted by different colors

**Fig. 4:**
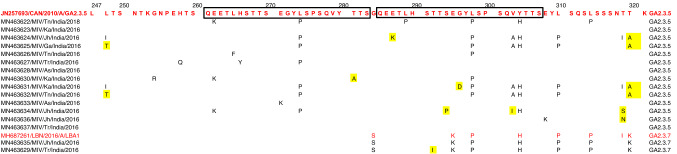
Multiple amino acid alignments of partial region of the second hypervariable region of G protein gene RSV-A subgroup sequences. Alignments are shown relative to the ON67-1210A sequence strain of NA1 genotype, aminoacid positions (247-321). Dots represent identical residues. Study sequences are in black font, whereas reference sequences in red font. Newly identified substitutions highlighted yellow in color

Phylogenetic analysis revealed that out of the 16 sequences of RSV-A subgroup, 14 sequences clustered with GA2.3.5 lineage of the GA2 genotype and 2 sequences clustered with GA2.3.7 lineage of GA2 genotype (Fig. 3), and the p-distance analysis revealed that the highest average intragenotypic p-distance was found for the GA1 genotype (p-distance = 0.062) and it was set as a threshold for the intergenotypic p-distance (Table 3). The lowest average intragenotypic p-distance was found for the GA3 genotype (*p*-distance = 0.044). The amino acid analysis in the partial region of the second hypervariable region of the G gene sequences revealed that all 16 study sequences had 24 amino acid duplication regions from 261 to 284 amino acid positions (Fig. 4). We found two substitutional changes at T292I and I319T in MN463629/MIV/Tr/India/2016, and a substitutional change at I319T in MN463635/MIV/Jh/India/2016 study sequences which were of GA2.3.7 lineage of GA2 genotype when compared with RSV-A/Lebanon/16LB22/2016 strain sequence of GA2.3.7 lineage of GA2 genotype sequence from 247 to 321 amino acid positions of G gene (Supplementary Table 3). The 14 study sequences that were of GA2.3.5 lineage of GA2 genotype were compared with the ON67-1210A strain sequence of GA2.3.5 lineage of GA2 genotype from 247 to 321 amino acid positions of the G gene. The amino acid substitutional changes were detected in 12 of these 14 sequences, whereas substitutional changes were not observed in the remaining two sequences (Supplementary Table 4). The aminoacid substitutional changes L248I (*n* = 2 sequences), G254R (*n* = 1), H258Q (*n* = 1), E262K (*n* = 3), L265F (*n* = 1), H266Y (*n* = 1), E271K (*n* = 2), L274P (*n* = 7), L289P (*n* = 1), L298P (*n* = 7), V303A (*n* = 4), Y304H (*n* = 6), E308K (*n* = 1), L314P (*n* = 1), and T319I (*n* = 2) were observed in our study sequences. New aminoacid substitutional changes L248T (*n* = 2 sequences), T281A (*n* = 1), E287K (*n* = 1), S294P (*n* = 1), G296D (*n* = 1), V303I (*n* = 1), T319S (*n* = 1), T319N (*n* = 1), and T320A (*n* = 4) were observed in our study sequences.

**Table 3:** Average intra and intergenotypic p-distance of RSV-A subgroup

	GA1	GA2	GA3
GA1	**0.062**		
GA2	0.166	**0.057**	
GA3	0.163	0.115	**0.044**

The intragenotypic p-distances are denoted in bold. The highest intragenotypic p-distance underlined

**Fig. 5:**
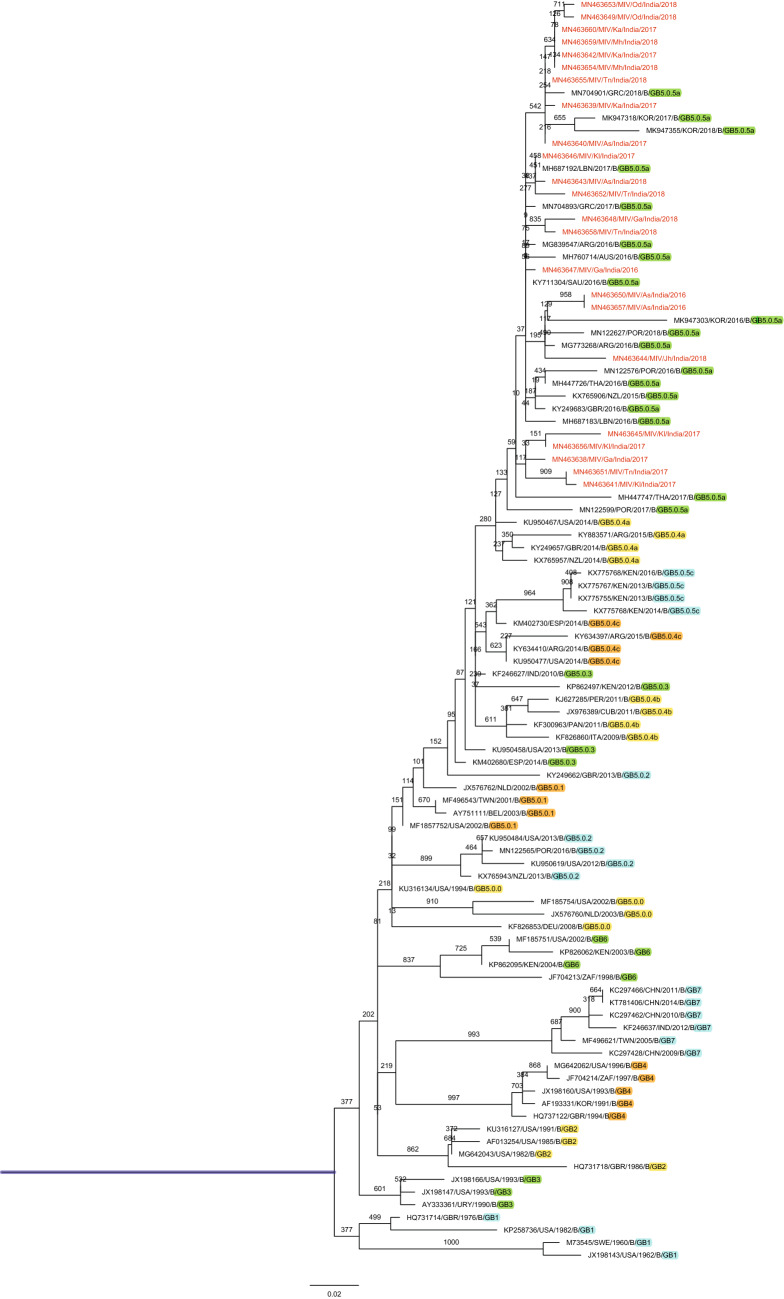
Mid-rooted phylogenetic tree of RSV-B subgroup. Mid-rooted phylogenetic tree of the second hypervariable region of the RSV-B G protein gene constructed by using Maximum Likelihood method, by HKY85 + G substitution model with 1000 bootstrap replicates in PhyML software. Study sequences are displayed in red. The reference sequences used to construct the tree were downloaded from the GenBank database. The two lettered words in the labels of the MIV isolates represent the state names, where: T* n* = Tamil Nadu, Ka = Karnataka, Jh = Jharkhand, Ga = Goa, Tr = Tripura, As = Assam, Kl = Kerala, Od = Odisha, and Mh = Maharashtra. The grid lines over the tree represent the patristic distance where the distance between each grid is 0.005. The names of the genotypes and lineages were highlighted by different colors

**Fig. 6:**
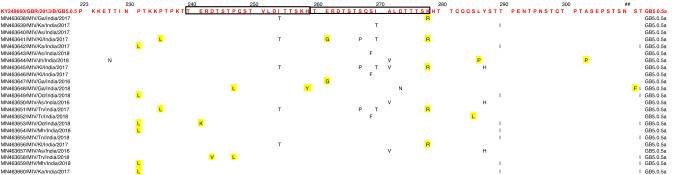
Multiple amino acid alignments of partial region of the second hypervariable region of G protein gene RSV-B subgroup sequences. Alignments are shown relative to the RSV-B/England583/2013 sequence strain of GB5.0.5a genotype, aminoacid positions (223-312). Dots represent identical residues. Study sequences are in black font, whereas reference sequence in red font. Newly identified substitutions highlighted yellow in color

Phylogenetic analysis revealed that all the 23 sequences of RSV-B subgroup clustered with GB5.0.5a lineage of the GB5 genotype (Fig. 5), the p-distance analysis was carried out for 6 genotypes (GB1, GB2, GB4, GB5, GB6, and GB7), the highest average intragenotypic p-distance was obtained for the GB1 genotype (*p*-distance = 0.070) and it was set as a threshold for the intergenotypic p-distance (Table 4). The lowest average intragenotypic p-distance was found for GB4 genotype (*p*-distance = 0.015). The amino acid analysis in the partial region of the second hypervariable region of the G gene sequences revealed that all 23 study sequences had 20 amino acid duplication regions from 240 to 269 amino acid positions (Fig. 6). These 23 study sequences were compared with the RSV-B/England583/2013 strain sequence of GB5.0.5a lineage of GB5 genotype from 223 to 312 amino acid positions of the G gene. The amino acid substitutional changes were detected in 22 of these 23 sequences, whereas substitutional changes were not observed in the remaining one sequence (Supplementary Table 5). The aminoacid substitutional changes T227N (*n* = 1 sequence), I254T (*n* = 5), S267P (*n* = 3), S269F (*n* = 3), I270T (*n* = 4), A271V (*n* = 3), D273N (*n* = 1), Y287H (*n* = 3), T290I (*n* = 8), and T312I (*n* = 8) were reported in our study [18, 26–28]. New aminoacid substitutional changes P231L (*n* = 6 sequences), P235L (*n* = 2), E241K (*n* = 1), D243V (*n* = 1), P247L (*n* = 2), H259Y (*n* = 1), E261G (*n* = 2), K278R (*n* = 5), S285L (*n* = 1 from Tripura), L286P (*n* = 1), A303P (*n* = 1), and S311F (*n* = 1) were observed in our study sequences. The old genotypes which were corresponding to respective newly reclassified genotypes are depicted in (Supplementary Table 6).

**Table 4:** Average intra and intergenotypic p-distance of RSV-B subgroup

	GB1	GB2	GB4	GB5	GB6	GB7
GB1	**0.070**					
GB2	0.091	**0.028**				
GB4	0.114	0.086	**0.015**			
GB5	0.100	0.074	0.103	**0.045**		
GB6	0.102	0.074	0.094	0.076	**0.036**	
GB7	0.103	0.081	0.108	0.099	0.103	**0.020**

The intragenotypic p-distances are denoted in bold. The highest intragenotypic p-distance underlined

## Discussion

RSV is one of the most common viral causes of acute respiratory illness (ARI) among infants and young children throughout the world [23]. In India, most of the molecular epidemiological studies were conducted in Assam, New Delhi, and Maharashtra to analyze the circulation and genetic diversity of RSV [5, 22, 23, 29, 30]. This study is the first of its kind which has covered 10 states, Karnataka, Kerala, Tamil Nadu, Assam, Goa, Gujarat, Maharashtra, Jharkhand, Tripura, and Odisha, and even different geographical locations that lie in southern, eastern, and western parts of India were analyzed from April 2016 to September 2018. Most of the molecular epidemiological studies of RSV usually focus only on children less than 5 years of age [1, 31]. In this study, we included cases from the age group 1–65 years. In the current study, phylogenetic analyses were performed based on the newly reclassified genotypes by Goya et al. [21]. Phylogenetic analysis of RSV-A subgroup revealed a novel genetic lineage GA2.3.7 for the first time in India to the best of our knowledge, including GA2.3.5 lineage of GA2 genotype. The distinct clade of GA2.3.7 lineage of GA2 genotype showed the patristic distance of approximately 0.120 when the distance was set as 0 (zero) at the ancestral node of 2.3.0 subgenotype, fulfilling the criteria to designate as a new genetic lineage having more than eight multiples of the patristic distance of 0.015 which was proposed by Goya et al. [21]. The patristic distance of 2.3.0 subgenotypes and further lineages in it is depicted in (Supplementary Fig. 1). GA2.3.7 lineage of GA2 genotype was first reported in 2016 in Lebanon with 72 bp nucleotide duplication similar to GA2.3.5 lineage of GA2 genotype with characteristic amino acid substitutions (G284S, E295K, Y304H, L314P, T319I, and P320K) [18]. In the current study sequences of GA2.3.7 lineage, a substitutional change I319T was observed in both the GA2.3.7 lineage study sequences, whereas T292I was observed in a sequence from Tripura when compared to RSV-A/Lebanon/16LB22/2016 strain sequence of GA2.3.7 lineage which was not observed in any of the GA2.3.7 lineage sequences that were reported by Abou-El-Hassan et al. [18]. We observed the circulation of GA2.3.7 lineage of GA2 genotype in 2016 among children in our study. The GA2.3.5 lineage of GA2 genotype RSV-A was first detected in Canada in 2011 and became the predominant genotype globally [32]. In the current study, we observed GA2.3.5 lineage of GA2 genotype as the predominant circulating genotype of RSV-A subgroup in the South, North, and Northeast regions of India during 2016–2018. Sahu et al. (Madhya Pradesh) reported the substitutional changes E262K, L274P, L298P, V303A, and L310P; Haider et al. (Delhi) reported the substitutional changes E262K, L274P, and L298P; Choudhary et al. (Maharashtra) reported the substitutional changes L274P, L298P, V303A, and L310P which were even observed in our study sequences [5, 23, 30]. The amino acid substitutional changes that were mentioned earlier in the results section (i.e., from L248I to T320A) were also reported by Cui et al., Ogunsemowo et al., Malasao et al., and Eshaghi et al. [32–35]. Few of the newly identified substitutional changes were also observed in our study sequences, but these changes did not constitute for defining these sequences as novel genotype based on the classification criteria defined by Goya et al. The circulating genotypes from different countries during the same period of our study (i.e., 2016, 2017, and 2018) were replicated same as our findings as GA2.3.5 lineage of GA2 genotype in South Korea, Portugal, Greece, Australia, Saudi Arabia, Lebanon, and Thailand [18, 26–28, 36–38]. In India, according to old classification previous studies reported circulation of GA2, GA5 genotypes (2001–2005), and ON1 genotype (2011–2015) in Delhi; GA5 and NA1 genotypes (2009–2012) in Assam; and NA1 lineage and ON1 genotypes in Pune (2009–2012), and NA1 and ON1 genotypes in Kerala (2012–2014) [14, 22, 23, 30, 39, 40]. At the time of preparing this manuscript, a publication by Broor et al. in 2019 related to the status of the molecular epidemiology of RSV in India was the latest information related to the circulated genotypes in India to the best of our knowledge [41].

Phylogenetic analysis of the RSV-B subgroup revealed GB5.0.5a lineage of GB5 genotype as the predominant circulating genotype in the South, North, and Northeast regions of India. GB5.0.5a was first detected in 2013 from England (Accession number: KY249660) from unpublished data with 60 bp nucleotide duplication region along with twelve signature amino acids [21]. Sahu et al. (Madhya Pradesh) reported the substitutional changes I254T, E261G, S267P, I270T, and Y287H; Haider et al. (Delhi) reported the substitutional changes T227N, E241K, I254T, S267P, I270T, A271V, Y287H, and T290I; Choudhary et al. (Maharashtra) reported the substitutional changes T227N, E261G, S267P, I270T, A271V, Y287H, and T290I which were even observed in our study sequences [5, 23, 42]. The amino acid substitutional changes that were mentioned earlier in the results section (i.e., from T227N to T312I) were also reported by Abou-El-Hassan et al., Tsergouli et al., Yun et al., and Al-Hassinah et al. [18, 26–28]. The circulating genotypes from different countries during the same period of our study (i.e., 2016, 2017, and 2018) were found to be of GB5.0.5a lineage of GB5 genotype in South Korea, Portugal, Greece, Australia, Saudi Arabia, Lebanon, and Thailand [18, 26–28, 36–38]. In India, according to the old classification, previous studies reported circulation of BA genotype (2001–2005), BA7, BA9, BA10, BA12 genotypes (2007–2010), SAB4, BA8, and BA9 genotypes (2011–2015) in Delhi; BA genotype (2005 -2008) in Kolkata; GB2, BA9, and BA12 genotypes (2009–2012) in Maharashtra; BA9 and BA10 genotypes (2012–2014) in Kerala [14, 22, 23, 39, 40, 42]. The p-distance analysis was carried out for 6 genotypes (GB1, GB2, GB4, GB5, GB6, and GB7) where 1 genotype (GB3) was excluded from the analysis as it had lacked signature amino acids significant for the genotypic determination in the second hypervariable region of the G gene [21].

Co-circulation of A and B subgroups were observed in this study, in 2016 with a predominance of RSV-A subgroup, whereas the RSV-B subgroup was predominant in 2017 and 2018. Similar studies with co-circulation of RSV-A and RSV-B were observed with RSV-B as a predominant subgroup in 2005–2006, whereas RSV-A in 2007–2008 in Kolkata [14]. RSV-B was observed to be predominant among co-circulating subgroups (2001–2002), RSV-A was observed to be predominant during (2002–2003, 2003–2004, and 2004–2005) in Delhi [22]. A study that analyzed the laboratory parameters of RSV infected individuals reported that the mean values of WBC count, ESR levels, and CRP levels as 9840 cells/mm^3^, 28 mm/h, and 27 mg/l, respectively [43]. In this study, we found the mean values of WBC count, ESR levels, and CRP levels were as follows: 6849 cells/mm^3^, 31.8 mm/h, and 15.6 mg/l respectively.

The circulation and spread of the GA2 genotype, and the GB5 genotype in India might be due to the change of the viral antigenic properties leading to the emergence of the new genotypes for evasion from the host immune response. The disappearance of old genotypes with the replacement of new genotypes of RSV might be because of the herd immunity developed by the individuals [44]. The development of a vaccine against circulating genotypes in a particular region can reduce the extent of the spread of RSV infection among children and old age.

This study has some limitations. Sequences of few samples were unable to obtain as the quality of the RNA was low as samples were from retrospective study. The newly identified substitutional changes need to be seen in further studies in other parts of India. The p-distance analysis was carried out for 6 genotypes (GB1, GB2, GB4, GB5, GB6, and GB7) where 1 genotype (GB3) was excluded from the analysis as it doesn’t have characteristic amino acid significant for the genotypic determination in the second hypervariable region of the G gene.

## Conclusions

GA2 genotype was the circulating genotype of the RSV-A subgroup, whereas GB5 was the circulating genotype of the RSV-B subgroup in the South, North, and Northeast regions of India during the period between 2016 and 2018. To the best of our knowledge, this is the first report to identify the GA2.3.7 lineage of GA2 genotype in India. RSV needs more surveillance studies on molecular epidemiology to get a better understanding of the circulating genotypes globally as we are observing mutations. Acute surveillance is required to know the predominance of RSV-A and RSV-B subgroups which may help in the implementation of the development of therapeutics or prevention against RSV infection. The consequences of the COVID19 pandemic and the restrictions applied to the movement of the population will be the most useful aspect leading to the decrease of the transfer of RSV infection among the population worldwide. If there is a possibility in maintaining the same social distancing norms even after the fall of COVID19 can lead to a massive decline in the infection rate of RSV and other transmissible diseases.

## Supplementary Information

Below is the link to the electronic supplementary material.

Supplementary file1 (DOCX 741 kb)

## Data Availability

Supporting data was made available in supplementary material 1 which can be made available for the reader.
