# Supplementary material for: Molecular epidemiology of respiratory syncytial virus among children and adults in India 2016 to 2018
Source: Virus Genes. 2021 Sep 15;57(6):489–501. doi: 10.1007/s11262-021-01859-4 (PMC8440155; doi:10.1007/s11262-021-01859-4)
Supplement: Supplementary file 1 — Supplementary file1 (DOCX 741 kb) [file 11262_2021_1859_MOESM1_ESM.docx]

**Molecular epidemiology of Respiratory Syncytial Virus among children and adults in India 2016 to 2018**

**Author names and affiliations:** Suresh S. Bandla^a^, Santhosha Devadiga^a^, Rushil Bhatt^a^, Oliver C Dsa^a,^ Arunkumar Govindakarnavar^a^@.

1. Manipal Institute of Virology (MIV), Manipal Academy of Higher Education (MAHE), Manipal, Karnataka-576104, India

**@Corresponding author**

Prof. Arunkumar Govindakarnavar, Ph.D.,

Manipal Institute of Virology,

Manipal Academy of Higher Education,

Manipal Karnataka State, India - 576 104.

Cell:+919845584163

Email: [arunviro@gmail.com](mailto:arunviro@gmail.com), [virology@manipal.edu](mailto:virology@manipal.edu)

# Currently working as a consultant with World Health Organization Country office for Nepal.

**Supplementary material 1**

**Supplementary table 1.** Clinical characteristics of RSV among adults and children

| Clinical symptom | N | Present | % | RSV (n=151) | | | P value |
| --- | --- | --- | --- | --- | --- | --- | --- |
|  |  |  |  | **Adult (>13 years)** | **Children (≤ 13 years)** | |  |
| Cough | 151 | 142 | 94 | 72 | | 70 | 0.778 |
| General weakness | 151 | 135 | 89.4 | 70 | | 65 | 0.703 |
| Coryza | 151 | 118 | 78.1 | 54 | | 64 | **0.015** |
| Headache | 151 | 111 | 73.5 | 67 | | 44 | **<0.001** |
| Myalgia | 151 | 102 | 67.5 | 60 | | 42 | **0.014** |
| Night sweats | 151 | 62 | 41.1 | 38 | | 24 | **0.035** |
| Sore throat | 151 | 60 | 29.7 | 40 | | 20 | **0.003** |
| Joint pain | 151 | 61 | 40.4 | 38 | | 23 | **0.036** |
| Breathlessness | 151 | 23 | 15.2 | 13 | | 10 | 0.613 |
| Chest pain | 151 | 20 | 13.2 | 17 | | 3 | **0.001** |

Supplementary table 2. Laboratory parameters of RSV cases among adult and children

| Laboratory parameters at admission |  | RSV | |
| --- | --- | --- | --- |
|  | **All** | **Adult (>13 years)** | **Children (≤13 years)** |
| Mean Age years (n=151) | 22.2 | 37.8 | 6.1 |
| Mean Total Leucocyte count /mm^3^ (n=111) | 6849 | 6194 | 7541.3 |
| Mean Differential Neutrophil percentage (n=105) | 61.3 | 65.3 | 53.2 |
| Mean Differential Lymphocyte percentage (n=105) | 31.6 | 28.1 | 39 |
| Mean Erythrocyte Sedimentation Rate mm/hr (n=22) | 31.8 | 37.5 | 24.9 |
| Mean C-Reactive protein levels mg/l (n=69) | 15.6 | 19.1 | 11.7 |

# Supplementary table 3. List of the substitutions detected among study sequences of GA2.3.7 lineage of GA2 genotype in comparison to RSVA/Lebanon/16LB22/2016 strain sequence of GA2.3.7 lineage of GA2 genotype

| Isolate name | No. substitutions | List of substitutions reported earlier | Newly identified substitution |
| --- | --- | --- | --- |
| MN463629/MIV/Tr/India/2016 | 2 | I319T | T292I |
| MN463635/MIV/Jh/India/2016 | 1 | I319T | - |

**Supplementary table 4.** List of the substitutions detected among study sequences of GA2.3.5 lineage of GA2 genotype in comparison to ON67-1210A strain sequence of GA2.3.5 lineage of GA2 genotype

| Isolate name | No. substitutions | List of substitutions reported earlier | Newly identified substitution |
| --- | --- | --- | --- |
| MN463636/MIV/Jh/India/2016 | 2 | E308K(1) | T319N |
| MN463634/MIV/Jh/India/2016 | 6 | E262K, L274P, Y304H(2,3) | S294P, V303I, T319S |
| MN463633/MIV/As/India/2016 | 1 | E271K(3) | - |
| MN463632/MIV/Tn/India/2016 | 6 | L274P, L298P, V303A, Y304H(2,3) | L248T, T320A |
| MN463631/MIV/Ka/India/2016 | 9 | L248I, L274P, L298P, V303A, Y304H, L310P, T319I(2,3) | G296D, T320A |
| MN463630/MIV/Ka/India/2016 | 4 | E262K, L298P(3) | G254R, T281A |
| MN463627/MIV/Tr/India/2016 | 3 | H258Q, H266Y, L274P(3,4) | - |
| MN463625/MIV/Ga/India/2016 | 7 | L274P, L298P, V303A, Y304H, L310P(2,3) | L248T, T320A |
| MN463624/MIV/Jh/India/2016 | 9 | L248I, L274P, L298P, V303A, Y304H, L310P, T319I(2,3) | E287K, T320A |
| MN463626/MIV/Tn/India/2016 | 2 | L265F, L298P(3) | - |
| MN463623/MIV/Ka/India/2016 | 1 | E271K(3) | - |
| MN463622/MIV/Tn/India/2018 | 6 | E262K, L274P, L289P, L298P, Y304H, L314P(2,3) |  |

#

# Supplementary table 5. List of the substitutions detected among study sequences of GB5.0.5a lineage of GB5 genotype in comparison to RSVB/England583/2013 strain sequence of GB5.0.5a lineage of GB5 genotype

| Isolate name | No. substitutions | List of substitutions reported earlier | Newly identified substitutions |
| --- | --- | --- | --- |
| MN463654/MIV/Mh/India/2018 | 2 | T290I(5) | P231L |
| MN463653/MIV/Od/India/2018 | 4 | T290I, T312I(5,6) | P231L, E241K |
| MN463649/MIV/Od/India/2018 | 3 | T290I,T312I(5,6) | P231L |
| MN463641/MIV/Kl/India/2017 | 6 | I254T, S267P, I270T(7) | P235L, E261G, K278R |
| MN463658/MIV/Tn/India/2018 | 3 | T312I(6) | D243V, P247L |
| MN463655/MIV/Tn/India/2018 | 2 | T290I, T312I(5,6) | - |
| MN463652/MIV/Tr/India/2018 | 2 | S269F(7) | S285L |
| MN463651/MIV/Tn/India/2017 | 5 | I254T, S267P, I270T(7) | P235L, K278R |
| MN463656/MIV/Kl/India/2017 | 2 | I254T(7) | K278R |
| MN463648/MIV/Ga/India/2018 | 5 | D273N, T312I(5–7) | P247L, H259Y, S311F |
| MN463646/MIV/Kl/India/2017 | 1 | S269F (7) | - |
| MN463645/MIV/Kl/India/2017 | 6 | I254T, S267P, I270T, A271V, Y287H (5,7) | K278R |
| MN463642/MIV/Ka/India/2017 | 3 | T290I, T312I(5,6) | P231L |
| MN463643/MIV/As/India/2018 | 1 | S269F(7) | - |
| MN463638/MIV/Ga/India/2017 | 3 | I254T (7) | K278R |
| MN463660/MIV/Ka/India/2017 | 3 | T290I, T312I(5,6) | P231L |
| MN463647/MIV/Ga/India/2016 | 1 | - | E261G |
| MN463639/MIV/Ka/India/2017 | 2 | I270T, T290I (5,7) | - |
| MN463659/MIV/Mh/India/2018 | 3 | T290I, T312I(5,6) | P231L |
| MN463657/MIV/As/India/2016 | 2 | A271V, Y287H (5,7) | - |
| MN463644/MIV/Jh/India/2018 | 4 | T227N, A271V (5,7,8) | L286P, A303P |
| MN463650/MIV/As/India/2016 | 2 | A271V, Y287H (5,7) | - |

**Supplementary table 6.** Newly reclassified genotypes in correspondence to old genotype classification based on identification of characteristic amino acids

| **RSV-A subgroup** | | **RSV-B subgroup** | |
| --- | --- | --- | --- |
| Old genotype classification | New reclassified genotype | Old genotype classification | New reclassified genotype |
| GA1 | GA1 | BA-CCA | GB5.0.5a |
|  |  | BA-CCB | GB5.0.5a |
| GA2 | GA2.3 | BA1 | GB5.0.1 |
|  |  | BA2 | GB5.0.1 |
| GA3 | GA2 | BA3 | GB5.0.1 |
|  |  | BA4 | GB5.0.1 |
| GA4 | GA3 | BA5 | GB5.0.1 |
|  |  | BA6 | GB5.0.1 |
| GA5 | GA3.0.3 | BA7 | GB5.0.2 |
|  |  | BA8 | GB5.0.2 |
| GA6 | GA2.1 | BA9 | GB5.0.5c |
|  |  | BA10 | GB5.0.2 |
| GA7 | GA2 | BA11 | GB5.0.4a |
|  |  | BA12 | GB5.0.2 |
| SAA1 | GA3 | BA14 | GB5.0.2 |
|  |  | BA-C | GB5.0.2 |
| SAA2 | GA2.3 | BA-LY | GB5.0.1 |
|  |  | GB1 | GB1 |
| NA1 | GA2.3.3 | GB2 | GB2 |
|  |  | GB3 | GB6 |
| NA2 | GA2.3.1 | GB4 | GB4 |
|  |  | GB5 | GB7 |
| NA3 | GA2.3.3 | GB6 | GB5 |
|  |  | GB7 | GB5 |
| NA4 | GA2.3 | GB8 | GB5 |
|  |  | GB9 | GB5 |
| ON1 | GA2.3.5 | GB10 | GB5 |
|  |  | GB11 | GB5 |
| CB-A | GA2.3.3 | GB12 | GB5 |
|  |  | GB13 | GB5.0.4b |
| TN-1 | GA2.3.4 | SAB1 | GB6 |
|  |  | SAB2 | GB5 |
| TN-2 | GA2.3.4 | SAB3 | GB5 |
|  |  | SAB4 | GB5 |
| LBA1 | GA2.3.7 | URU-1 | GB5 |
|  |  | CB-1 | GB7 |
| LBA2 | GA2.3.4 | THB | GB7 |


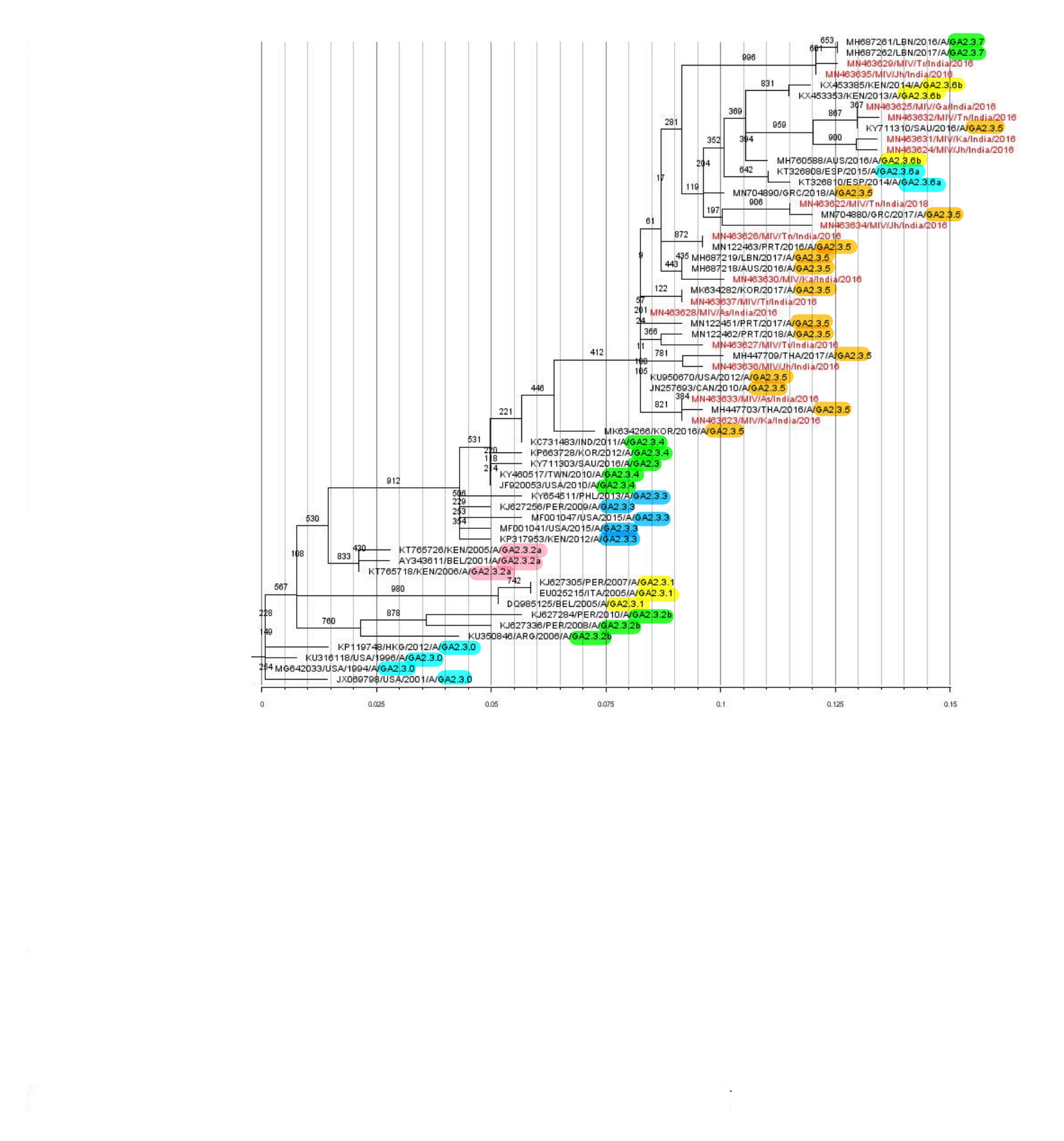


Supplementary Fig.1: The figure shows the lineages of the GA 2.3.0 subgenotype of GA2 genotype. The grid lines over the tree represent the patristic distance where the distance between each grid is 0.005. The names of the lineages were highlighted by different colours.

References:

1. Cui G, Qian Y, Zhu R, Deng J, Zhao L, Sun Y, et al. Emerging human respiratory syncytial virus genotype ON1 found in infants with pneumonia in Beijing, China. Emerg Microbes Infect. 2013 Apr;2(4):e22.

2. Ogunsemowo O, Olaleye DO, Odaibo GN. Genetic diversity of human respiratory syncytial virus circulating among children in Ibadan, Nigeria. Cormier SA, editor. PLOS ONE. 2018 Jan 23;13(1):e0191494.

3. Malasao R, Okamoto M, Chaimongkol N, Imamura T, Tohma K, Dapat I, et al. Molecular Characterization of Human Respiratory Syncytial Virus in the Philippines, 2012-2013. Turner SJ, editor. PLOS ONE. 2015 Nov 5;10(11):e0142192.

4. Eshaghi A, Duvvuri VR, Lai R, Nadarajah JT, Li A, Patel SN, et al. Genetic Variability of Human Respiratory Syncytial Virus A Strains Circulating in Ontario: A Novel Genotype with a 72 Nucleotide G Gene Duplication. Khudyakov YE, editor. PLoS ONE. 2012 Mar 28;7(3):e32807.

5. Abou-El-Hassan H, Massaad E, Soudani N, Assaf-Casals A, Shaker R, Lteif Khoury M, et al. Detection of ON1 and novel genotypes of human respiratory syncytial virus and emergence of palivizumab resistance in Lebanon. Russell CJ, editor. PLOS ONE. 2019 Feb 21;14(2):e0212687.

6. Tsergouli K, Pappa S, Haidopoulou K, Gogou M, Giannopoulos A, Papa A. Respiratory Syncytial Virus in Greece, 2016–2018. Intervirology. 2019;62(5–6):210–5.

7. Yun KW, Choi EH, Lee HJ. Molecular epidemiology of respiratory syncytial virus for 28 consecutive seasons (1990-2018) and genetic variability of the duplication region in the G gene of genotypes ON1 and BA in South Korea. Arch Virol. 2020 May;165(5):1069–77.

8. Al-Hassinah S, Parveen S, Somily AM, AlSaadi MM, Alamery SF, Haq SH, et al. Evolutionary analysis of the ON1 genotype of subtype a respiratory syncytial virus in Riyadh during 2008–16. Infect Genet Evol. 2020 Apr;79:104153.
